# Segmentation-Free Preoperative 3D MRI Classification of Low-Grade Versus High-Grade Glioma Using Task-Oriented Neural Architecture Search

**DOI:** 10.3390/jimaging12060254

**Published:** 2026-06-08

**Authors:** Christos Ch. Andrianos, Spiros A. Kostopoulos, Ioannis K. Kalatzis, Dimitris Th. Glotsos, Pantelis A. Asvestas, Dionisis A. Cavouras, Emmanouil I. Athanasiadis

**Affiliations:** Medical Image and Signal Processing Laboratory, Department of Biomedical Engineering, University of West Attica, 12241 Athens, Greece; chandrianos@uniwa.gr (C.C.A.); skostopoulos@uniwa.gr (S.A.K.); ikalatzis@uniwa.gr (I.K.K.); dimglo@uniwa.gr (D.T.G.); pasv@uniwa.gr (P.A.A.); cavouras@uniwa.gr (D.A.C.)

**Keywords:** glioma, 3D convolutional neural network, segmentation-free classification, neural architecture search, explainable artificial intelligence

## Abstract

Gliomas constitute the majority of primary brain tumors, and accurate diagnosis through MRI is essential for patient management. Existing computer-aided diagnosis approaches frequently rely on tumor segmentation frameworks. In this study, a segmentation-independent framework for volumetric low-grade versus high-grade glioma (LGG/HGG) classification is proposed using a Convolutional Neural Network (CNN) designed through task-oriented Neural Architecture Search (NAS). The proposed method was evaluated on a multi-center dataset comprising 1194 patients with pre-operative MRI scans, including T1-CE and FLAIR sequences from four publicly available cohorts. NAS was conducted within a controlled search space to optimize a 3D U-Net–based backbone using Tree-structured Parzen Estimator (TPE) combined with Hyperband pruning. The optimized backbone was enhanced with residual connections and Squeeze-and-Excitation (SE) attention mechanisms to improve feature representation and training stability. Internal validation employed repeated 5-fold cross-validation across all four multi-center datasets. An external experiment used REMBRANDT as a test cohort (49 LGG, 19 HGG). The proposed model achieved 88.25% internal accuracy and 75.51% external accuracy (macro-F1: 87.37% internal, 73.77% external), outperforming benchmark 3D CNNs. Explainable Artificial Intelligence (XAI) analysis based on Grad-CAM revealed robust tumor localization without segmentation supervision, validated against available ground-truth masks. Additional experiments demonstrated the model’s generalization capacity, achieving 89.51% accuracy for IDH mutation prediction and 78.74% for multi-grade classification.

## 1. Introduction

Term glioma refers to a heterogeneous group of primary brain tumors that arise within Central Nervous System (CNS). The World Health Organization (WHO) classification categorizes these tumors into four grades (G1–4), based on histopathological characteristics and genetic alterations, aiming to determine tumor malignancy and guide patient management. In adults, most gliomas belong to the category of adult-type diffuse gliomas, distinct from pediatric-type gliomas in the current WHO classification. An important factor in modern glioma classification is the isocitrate dehydrogenase (IDH) mutation status, constituting a key biomarker for diagnostic and prognostic assessment [1,2].

In Computer-Aided Diagnosis (CAD) studies, glioma grading is frequently operationalized as low-grade glioma (LGG; G2–3) versus high-grade glioma (HGG; G4). This distinction reflects clinical practice, where G4 tumors represent the most aggressive and frequently observed malignant gliomas. Additionally, the presence of IDH mutation in G3 tumors has challenged the traditional definition of high-grade gliomas [3,4,5].

Recent studies have explored a wide range of deep learning architectures to classify gliomas, including CNNs, Vision Transformers (ViTs), and hybrid approaches. These methods have been increasingly adopted within multi-task learning frameworks to address both segmentation and classification tasks [6]. Early efforts in this direction conclude the utilization of sequential pipelines. For instance, Decuyper et al. [7] developed an automated framework that initially employs a 3D U-Net trained on the BraTS 2019 dataset to perform multi-modal MRI volumetric segmentation. Subsequently, the system extracts 3D regions of interest (ROIs) from an expanded dataset that includes cases from TCIA as well as an independent cohort obtained from Ghent University Hospital (GUH). The extracted ROIs are then processed by a ResNet-based CNN architecture within a multi-task manner, to simultaneously predict tumor grade, IDH mutation, and 1p19q co-deletion status.

More recently, Farahani et al. [8] proposed a multi-task framework (MTS-UNET), capable of simultaneously performing glioma segmentation, molecular subtyping, and histological grading from multimodal MRI data, acquired from multiple public datasets. Their approach is based on a pretrained SWIN-UNETR architecture (BrainSegFounder). The model incorporates a Tumor-Aware Feature Encoding (TAFE) module that leverages supervision from the segmentation branch to extract multi-scale, tumor-specific features for classification. Additionally, a Cross-Modality Differential (CMD) module captures imaging patterns such as the T2–FLAIR mismatch sign associated with IDH mutation.

Despite their promising performance, segmentation-dependent frameworks introduce additional computational complexity and increase processing time within the analysis workflow. Moreover, the heterogeneous nature of gliomas and the presence of ambiguous tumor boundaries in MRI images may negatively affect segmentation quality. In many cases, lesion regions are defined through relative intensity differences with adjacent tissues, resulting in variability even among expert manual annotations. Such inaccuracies may propagate errors to downstream classification tasks. In addition, developing these models typically requires expert-annotated ground-truth masks, the creation of which is both time-consuming and resource-intensive. Consequently, performing direct volumetric classification remains challenging in deep learning, particularly because the tumor-related regions consist of a small portion of MRI volume compared to normal tissue and background [9,10].

To reduce the dependency on explicit tumor segmentation, several studies have proposed segmentation-free approaches for glioma classification. A notable example is the cooperative multi-task framework (CMTLNet) proposed by Chen et al. [11], which aims to simultaneously predict glioma grade and molecular subtypes. The method introduces a segmentation-free tumor feature perception (SFTFP) module that learns tumor-aware representations through a classification-based approach. Based on the multi-scale tumor-aware features extracted by SFTFP, a task-common feature extraction (CFE) module learns shared representations across tasks, while a task-unique feature extraction (UFE) network captures task-specific features for each objective. Although the method is segmentation-free during inference, training remains reliant on voxel-level tumor masks to guide the network to distinguish between tumor-related and unrelated regions.

Within this context, segmentation-free volumetric classification remains relatively underexplored in the literature. An earlier study by Chakrabarty et al. [12] investigated a direct volumetric MRI classification framework. The task concerned distinguishing between healthy subjects from multiple brain tumor categories in a seven-class problem, which also included the LGG/HGG classification task. The authors proposed a CNN model inspired from a segmentation architecture by retaining only the context-encoding path, and adding a classification head. However, the model was inspired by a successful segmentation architecture, the final classification network was obtained through manual architectural adaptation rather than systematic optimization or architecture search process. Consequently, the resulting architecture may not necessarily represent an optimal configuration for the classification task.

Based on the described background, the main contributions of this study can be summarized as follows:

- A customized 3D CNN framework for pre-operative LGG/HGG classification from MRI data, without requiring an explicit tumor segmentation step.
- Integration of Neural Architecture Search (NAS) within a controlled search space inspired by well-performing segmentation architectures, enabling the automated discovery of an optimized backbone network for volumetric classification.
- Architectural enhancements exploration through the incorporation of residual connections and Squeeze-and-Excitation attention blocks to improve feature representation and network performance.
- Explainable Artificial Intelligence (XAI) analysis using Grad-CAM to provide qualitative visualizations of the model’s decision regions, followed by a quantitative agreement analysis between the activation maps and the available ground-truth tumor masks.
- Evaluation of the proposed framework on additional clinically relevant tasks, including IDH mutation status prediction and multi-grade glioma classification, to further investigate its robustness and generalization capability.

## 2. Materials and Methods

### 2.1. Data Collection

Four retrospective multi-center, publicly available MRI brain tumor collections were utilized to achieve the goal of this study. These datasets met the required primary inclusion criteria, specifically the provision of histologically confirmed glioma diagnosis according to the WHO classification system, as well as the availability of annotated pre-operative MRI sequence information.

Three of the selected collections were derived from The Cancer Imaging Archive (TCIA) [13]. The Repository of Molecular Brain Neoplasia Data (REMBRANDT) comprises 130 LGG and HGG patients [14]. The Cancer Genome Atlas Low Grade Glioma (TCGA-LGG) collection provides 199 cases diagnosed with grade 2 and 3 tumors [15]. The University of California San Francisco Preoperative Diffuse Glioma (UCSF-PDGM) MRI image collection consists of 495 subjects and incorporates imaging data from benchmarking initiatives such as the International Brain Tumor Segmentation (BraTS) challenge [10,16]. An additional independent cohort, the Erasmus Glioma Database (EGD) including 774 glioma MRI scans [17]. Although selected images derived from several collections originated from TCIA-related repositories or incorporated BraTS-associated imaging initiatives, all cohorts were examined for potential patient duplication before inclusion to eliminate effects of potential data leakage and unclear data separation. No overlapping patient identifiers were detected across the selected datasets.

Following data acquisition, a structured evaluation was conducted to define the final patient cohort for the system development. The exclusion process was performed in two sequential stages. In Stage 1, cases without available histopathological grade information were excluded, since tumor grade represented the ground-truth label for the supervised LGG versus HGG classification task.

In Stage 2, the remaining cases were assessed according to MRI sequence availability and image quality. T1-CE and FLAIR constituted the common denominator across the four collections and were therefore selected as the target sequences, as REMBRANDT and TCGA-LGG do not consistently provide complete multi-sequence MRI volumes. Cases were excluded when neither of the two selected sequences was available, whereas subjects with only one available sequence were retained.

**Figure 1:**
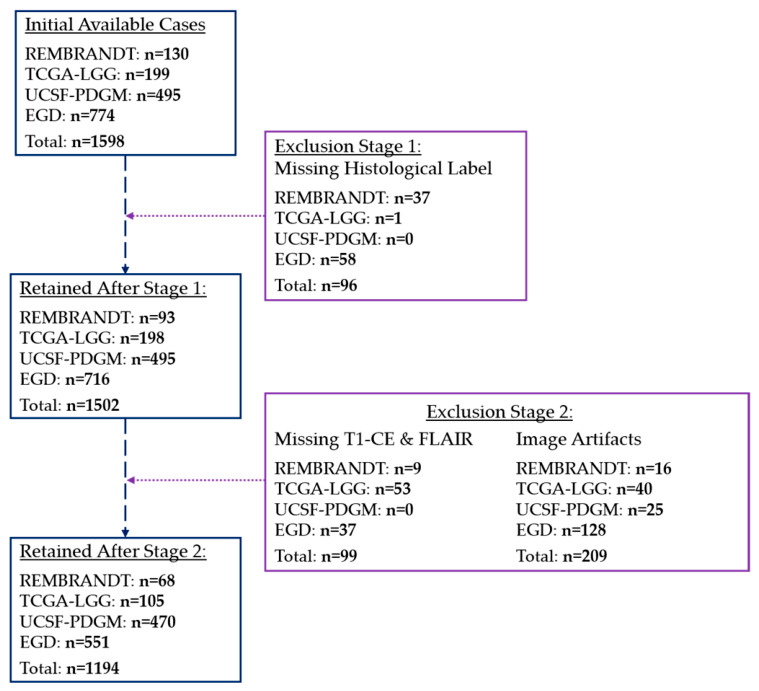
Flowchart of the two-stage patient inclusion and exclusion process across the four public MRI glioma collections. The number of excluded cases is reported for each exclusion criterion and data collection. Blue boxes represent patient cohorts retained after each selection stage, whereas purple boxes indicate excluded cases and the corresponding exclusion criteria.

MRI scans presenting severe artifacts were further excluded following manual visual quality control. Severe artifacts were defined as degradations that substantially impaired anatomical interpretation of the brain or tumor region across the 3D volume, including pronounced motion artifacts, acquisition-related distortions, corrupted or incomplete volumes, major signal dropout, or marked inter-slice inconsistencies. This step is particularly important when employing 3D CNNs, where spatial inconsistencies between slices due to quality degradation may influence the global volumetric representation and affect model performance [18,19]. The complete patient-level inclusion and exclusion process is summarized in Figure 1, including the corresponding number of excluded cases per criterion and per data collection.

After applying the inclusion criteria, a total of 1194 patients constituted the final dataset. Table 1 summarizes the distribution of cases according to tumor grading and additionally the availability of molecular IDH status within subsets of the UCSF-PDGM and EGD collections. Within the final cohort, 1019 patients included both sequences, while 60 patients contained only T1-CE and 115 only FLAIR.

Patients included in this study were adults, with an age range of 19 to 86 years. MRI examinations were acquired across multiple institutions, with a variety of scanner manufacturers, including Philips, Siemens and GE Healthcare, encompassing various system models. MRI field strengths ranged from 1 T to 3 T, with slice thickness varying between 1.9 mm and 6.0 mm. In total, these characteristics highlight the heterogeneous nature of the dataset and mitigate potential scanner-related bias.

### 2.2. Data Pre-Processing

**Figure 2:**
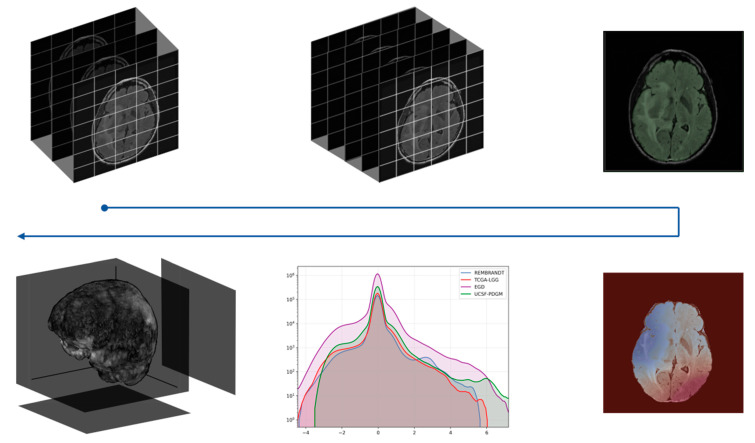
Schematic representation of pre-processing workflow applied prior to model training. Raw data underwent interpolation, skull-stripping, N4 bias field correction, z-score normalization and padding or cropping to achieve common spatial shape without altering anatomical information.

According to the literature, several preprocessing stages are commonly applied prior to CNN input, particularly in the context of 3D volumetric data, for segmentation or classification tasks [20,21,22,23]. In the present research, data were obtained from four independent collections that had undergone different procedures prior to public release. Therefore, a common pre-processing pipeline was implemented to ensure consistency across data cohorts. The overall preprocessing workflow is illustrated in Figure 2 and includes volume interpolation, skull-stripping, N4 bias field correction, z-score normalization and spatial resizing to set common shape.

Initially, the REMBRANDT and TCGA-LGG datasets provided raw axial DICOM data which were converted into 3D volumes in NIfTI format. The next step was to apply third-order spline interpolation to resample images, adjusting voxel spacing equal to 1 mm^3^, providing isotropic resolution. Compared with lower-order resampling techniques, cubic B-spline generates a third-order continuous approximation grid that balances discrete voxel intensity values, resulting in smoother transitions. This approach is preferred due to its suitability for both upsampling and downsampling procedures [24,25].

A crucial preprocessing step was the brain extraction that was performed on the volumes subsequently. At this stage, the EGD dataset was also included in the pipeline as it had already been provided in isotropic 1 mm^3^ resolution. Therefore, redundant interpolation was intentionally avoided to minimize unnecessary resampling effects and preserve the original volumetric information. The presence of non-brain tissue in MRI volumes is undesirable, as it introduces irrelevant signal from surrounding anatomical structures, such as the skull, eyes and tongue. To address this, a deep learning-based approach, SynthStrip was employed. SynthStrip is a multimodal pre-trained CNN that has demonstrated strong generalization across diverse MRI datasets, and improved performance over traditional and deep learning-based skull-stripping techniques [26].

Subsequently, N4 bias field correction was applied to remove MRI signal nonuniformity, with the UCSF-PDGM dataset entering the pipeline at this stage, as it had already undergone the preceding preprocessing steps. We skipped reapplying these preprocessing operations to avoid degrading the data or introducing artifacts. This artifact represents a low-frequency multiplicative intensity variation, arising from magnetic field inhomogeneities and anatomical factors related to MRI acquisition [27]. Given the integration of multi-center data from four independent collections, bias field correction was considered essential to reduce inter-scanner intensity variability and enhance the generalization capability of the CNN model. A two-stage strategy was implemented, in which images were initially processed at half spatial resolution for three sets of 50 iterations, followed by an additional 50 iterations at full resolution [28]. An Otsu-derived image mask (threshold = 200) was incorporated during correction to constrain the bias field estimation to relevant tissue regions and avoid altering meaningful anatomical information.

After bias field correction, z-score normalization was applied as a standardization technique to ensure intensity comparability across all volumes. Each 3D patient volume was independently normalized using non-zero voxels only, such that the resulting intensities had zero mean and unit standard deviation [29]. To reduce the influence of extreme intensity values, the 0.5th and 99.5th percentiles were excluded prior to normalization.

After completion of the aforementioned steps, all MRI volumes shared a common voxel spacing and spatial resolution. However, they differed in spatial dimensions and number of slices, preventing their direct input into a CNN architecture that requires a fixed global shape. For this reason, dimensional analysis and exclusion of extreme outliers followed, and a common spatial size of 240 × 240 × 180 (x, y, z) was selected, corresponding to the maximum dimensions to which all volumes were equal or smaller along each axis. Symmetric cropping or zero-padding was subsequently applied to standardize all volumes to this fixed shape, as reported not to negatively impact classification performance [30]. This approach preserved the original spatial information, while avoiding additional resampling that could introduce geometric distortion. Finally, all volumes were reoriented to a common RAS coordinate system to ensure consistent anatomical alignment across datasets.

Following preprocessing and volumetric preparation, all 2213 MRI volumes (T1-CE and FLAIR) underwent visual quality-control inspection to identify corrupted outputs, severe artifacts, or anatomically inconsistent preprocessing results before model training. No significant preprocessing failures or extreme anatomical distortions were observed, and therefore no further patient exclusion was required. Additionally, in cases where one imaging modality (T1-CE or FLAIR) was unavailable, the corresponding input channel was filled with zero values to preserve a consistent two-channel input representation while retaining all available patients.

### 2.3. Adoption of a 3D U-Net-Based Backbone

In medical imaging, CNNs have been widely adopted due to their ability to learn hierarchical feature representations directly from imaging data, achieving high classification performance across several tasks. In volumetric imaging, 3D CNNs offer a significant advantage by capturing spatial dependencies across all anatomical planes, thereby preserving inter-slice contextual information [31]. This characteristic is particularly important in context of MRI examinations, which are inherently three-dimensional by nature. In contrast, although 2D CNN approaches require lower computational cost, they process each volume slice independently, potentially overlooking volumetric relationships within the data. However, the volumetric nature of 3D CNNs increases architectural complexity, rendering design and optimization more sensitive and challenging.

To address this challenge, structured architectures such as the 3D U-Net have been widely adopted in volumetric medical imaging. The 3D U-Net represents a volumetric extension of the original 2D U-Net framework, incorporating three-dimensional convolutional layers to enable spatial feature learning [32]. It comprises a contracting path, known as the encoder, responsible for hierarchical feature extraction from the input images, through progressive spatial downsampling. Correspondingly, the expanding path is referred to as the decoder, designed for spatial reconstruction in segmentation tasks.

In the present study, the encoder component of the 3D U-Net was adopted as the core backbone element due to its ability to extract multi-scale feature representations and implicitly localize discriminative regions, such as the brain tumor area, within the full volume. This capability arises from the progressive doubling of convolutional filters across successive blocks, which increases representational depth as spatial resolution is gradually reduced through pooling. These architectural characteristics provide a robust baseline for segmentation-free volumetric classification, forming a structured starting point for subsequent task-oriented architectural optimization. Unlike segmentation tasks, volumetric classification does not require spatial reconstruction of the input representation. Therefore, only the encoder pathway was retained, allowing the network to focus exclusively on discriminative feature extraction while reducing unnecessary architectural complexity associated with the decoder component.

### 2.4. Task-Oriented Neural Architecture Search

Neural Architecture Search (NAS) is an Automated Machine Learning (AutoML) field, that systematically explores predefined neural network architectures to identify high-performing structures. The NAS method has shown performance improvement, in comparison with manually designed architectures on a variety of tasks. NAS shares conceptual overlap with Hyperparameter Optimization (HPO), indicating a broader meaning. Key parts of NAS are the search space, the search strategy, and the performance estimation protocol [33].

In previous 2D glioma classification studies, broad HPO schemes have been employed, to optimize architectural and training hyperparameters across extensive search ranges [34,35]. However, in volumetric 3D settings, the increased number of parameters and computational requirements make exhaustive architectural exploration significantly more challenging. For this reason, the present study adopted a constrained NAS framework based on a 3D U-Net encoder backbone, while maintaining fixed training and regularization hyperparameters to isolate architectural effects. Rather than exhaustively exploring arbitrary configurations, the search space was intentionally designed to focus on meaningful architectural variations for volumetric glioma classification. The explored parameters were selected to balance representational capacity, spatial information preservation, and computational feasibility within memory-intensive 3D CNN environments.

Specifically, a variety of encoder configurations were explored, with the corresponding ranges summarized in Table 2. Architectural depth varied between 3 and 6 convolutional blocks, while the initial number of base filters was defined categorically as either 16 or 32. Consistent with U-Net’s progressive filter doubling strategy, these options determined the encoder’s representational capacity. The number of convolutional layers per block ranged between 1 and 3, to investigate the impact of increased intra-block feature refinement on performance. Early-stage pooling configuration was additionally explored to evaluate the effect of preserving spatial resolution. The transition to the classification head was depth-dependent, with flattening used for three-block architectures and global pooling applied in deeper models to control parameter growth. Normalization types within blocks were also included in the search space.

To isolate architectural effects, all training hyperparameters were kept fixed, with the Adam optimizer and a learning rate of 1 × 10^−4^ applied throughout the search process. Under these constraints, the defined architectural search space comprised **2016** possible configuration combinations.

The search strategy defines how the architectural space is explored and optimized. To identify high-performing configurations the Tree-structured Parzen Estimator (TPE) sampler was implemented [36]. TPE models the probability distributions of promising and non-promising configurations, enabling efficient exploration of high-dimensional and categorical search spaces. To further enhance computational allocation, the Hyperband pruning strategy was integrated, allowing early termination of underperforming trials through a successive halving mechanism [37]. Given the computational demands of repeated 3D CNN training on volumetric MRI data, the search budget was constrained to 50 trials as a balance between computational feasibility and search-space exploration [38].

Regarding performance estimation, each structural combination proposed by TPE was evaluated under the combination of Hyperband and Early Stopping [37,39]. Hyperband monitored validation accuracy to identify unpromising combinations and prune them accordingly. In parallel, early stopping was applied based on validation loss, with a patience of three epochs, to prevent overfitting and reduce unnecessary computational cost. During the NAS phase, model performance was estimated using a random patient-level 75:25 hold-out split for the purpose of backbone CNN selection, providing an initial indication of architectural effectiveness, while ensuring efficient allocation of computational resources.

### 2.5. Architectural Enhancement of the Optimized Backbone

Following the determination of the backbone CNN through NAS, additional architectural refinements were explored to further enhance model performance. The optimized backbone consisted of a sequential convolutional architecture, within which residual learning was incorporated. Residual learning addresses the degradation effects, especially in deep volumetric networks. This mechanism employs shortcuts, also known as skip connections, that add the input of each block to its transformed output, facilitating improved gradient flow without increasing model complexity [40]. In the present architecture, residual connections were integrated within the encoder blocks, and when channel depth increased, 1 × 1 × 1 convolutional projection shortcuts were employed to ensure dimensional compatibility between feature maps.

In addition to residual learning, channel-wise attention mechanisms were incorporated through the Squeeze-and-Excitation (SE) blocks. The SE mechanism performs adaptive feature recalibration by emphasizing informative, class-discriminative features while suppressing less relevant ones. During the squeeze phase, global average pooling is applied to aggregate the spatial information of each convolutional feature map into a single representative value. In the subsequent excitation phase, two fully connected layers generate channel-wise modulation weights, which are applied multiplicatively to the input feature maps [41]. In context of this study, SE modules were integrated within each encoder block, after the application of conv and before pooling, positioned after the convolutional operations and prior to residual summation, allowing channel recalibration before feature fusion.

The overall output of each enhanced encoder block can be expressed as:

*Y* = *F(X)* ⋅ *SE(X)* + *X*

where *X* denotes the block input, *F(X)* represents the convolutional transformation, and *SE(X)* corresponds to recalibration weights generated by SE mechanism.

### 2.6. Explainability Assessment via Grad-CAM and Localization Metrics

Beyond performance evaluation of the proposed model, assessing the spatial basis of its predictions is essential for clinical applicability. By nature, deep neural networks are described as “black boxes” due to their limited interpretability, as internal feature transformations are not directly observable. To address this limitation, Explainable Artificial Intelligence (XAI) techniques have been developed to provide post hoc insight into model decision mechanisms [42]. Furthermore, current regulatory frameworks, including the European Union Artificial Intelligence Act (AIA), emphasize the importance of transparency and explainability in clinical decision support systems [43].

Considering the above, Gradient-weighted Class Activation Mapping (Grad-CAM) was employed to enhance model transparency and assess the spatial regions contributing to the network’s predictions. According to the Grad-CAM framework, an effective visual explanation should be class-discriminative, meaning that it localizes regions relevant to the predicted category [44]. In the context of glioma classification, this corresponds to highlighting tumor tissue rather than normal brain structures or background regions.

In the present study, Grad-CAM was not limited to qualitative visualization. High-contribution voxels (values exceeding the 90th percentile of the activation map) were extracted and quantitatively compared against available ground-truth tumor masks from the EGD and UCSF-PDGM datasets. Spatial agreement between activation maps and tumor masks was measured through Dice coefficient and Intersection over Union (IoU) defined respectively as:

$$Dice=\frac{2\;\left|A\;\cap \;B\right|}{\left|A\right|\;+\;\left|B\right|}$$

$$IoU=\frac{\left|A\;\cap \;B\right|}{\left|A\;\cup \;B\right|}$$

where *A* denotes the thresholded Grad-CAM region and *B* the ground-truth mask.

In addition to overlap-based metrics, an Energy Concentration Ratio was computed to quantify the distribution of Grad-CAM activations inside versus outside tumor regions:

$$ECR=\frac{\mu_{inside}}{\mu_{outside}},\;{\mathrm{ECR}}_{\mathrm{norm}}\;=\;\frac{ECR}{ECR+1}$$

where *μ_inside_* and *μ_outside_* represent the mean activation values within and outside the tumor mask, respectively. The ratio was then normalized (ECR_norm_) to the range (0, 1) to facilitate stable comparison across cases.

Beyond global similarity metrics, the availability of subregion masks in the UCSF-PDGM dataset enabled a more detailed contribution analysis. Grad-CAM activations were quantified within distinct tumor subregions, including necrosis, edema and enhancing tumor. Necrosis, also termed the non-enhancing tumor core, comprises necrotic and pre-necrotic regions, while peritumoral edema represents the infiltrative tumor margin and appears hyperintense on FLAIR imaging. The contrast-enhancing tumor corresponds to regions demonstrating contrast uptake on T1-CE sequences due to blood–brain barrier disruption. Notably, the presence of enhancing tumor and necrosis is strongly associated with HGG diagnosis [45]. The relative percentage of Grad-CAM activation within each subregion was computed to assess whether model predictions were preferentially driven by clinically relevant tumor characteristics.

### 2.7. Training Protocol and Performance Evaluation

Following the determination of the proposed architecture, a structured evaluation protocol was adopted to assess model generalization, stability, and potential overfitting. Repeated K-fold Cross-Validation, a widely adopted resampling strategy in which the dataset is partitioned into *k* equally sized subsets, was implemented to estimate model performance. The model is trained and validated k times, each time using a different fold for validation and the remaining folds for training. Repeating this procedure with different random splits reduces variance and provides a more robust estimation of true performance [46].

In the present study, a five-fold cross-validation scheme was applied and repeated across three different seeds, resulting in a total of 15 independent training runs. For each repetition, the folds were stratified according to the tumor-grade label to preserve the LGG/HGG class distribution across training and validation sets. Each run was conducted for up to 25 training epochs with a batch size of 5, representing the maximum feasible size under the memory demands of dual-channel volumetric 3D MRI processing in the present study. Early stopping was applied based on validation loss using a patience of 7 epochs. Sparse categorical cross-entropy was utilized as the loss function. Given the moderate class imbalance (442 LGG vs. 752 HGG cases), no explicit class balancing strategy or data augmentation techniques were applied during training. This design choice was intended to isolate the effects of the NAS-derived architectural optimization under standardized training conditions, while minimizing anatomically unrealistic variations in volumetric MRI data. Evaluation metrics included Accuracy (ACC), macro-averaged F1-score, Area Under the Curve (AUC), and Matthews Correlation Coefficient (MCC). All experiments were conducted on a High-Performance Computing (HPC) system, utilizing a single NVIDIA A100 GPU with 40 GB VRAM.

## 3. Results

### 3.1. Final NAS-Selected 3D CNN Architecture

The final 3D segmentation-free CNN backbone architecture for MRI-based glioma classification was selected from the three highest-performing candidate models identified during the NAS phase, summarized in Table A1. To ensure that model selection was not influenced by a favorable data split resulting from the hold-out strategy used in NAS, these architectures were subsequently re-evaluated using repeated five-fold cross-validation.

**Figure 3:**
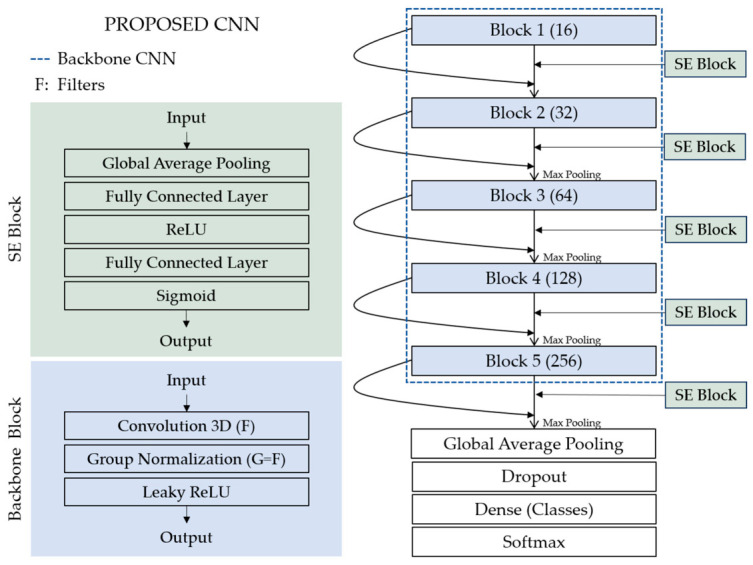
Schematic representation of the proposed 3D CNN architecture determined through Neural Architecture Search (NAS). The backbone consists of five hierarchical convolutional blocks enhanced with residual connections and squeeze-and-excitation (SE) attention modules. Blue elements denote the convolutional backbone, whereas green elements represent SE attention modules.

The proposed CNN architecture is illustrated in Figure 3. The final model comprises approximately 1.3 million trainable parameters, resulting in a relatively lightweight 3D design for volumetric MRI analysis. It consists of an optimized 3D convolutional backbone enhanced with residual connections and SE attention modules. The backbone structure includes five hierarchical blocks, each containing a single 3D convolutional layer followed by Group Normalization and LeakyReLU activation components, as depicted in the blue region of Figure 3. In the selected configuration, the number of groups in the Group Normalization layers was set equal to the number of feature channels, resulting in channel-wise normalization. The network begins with 16 filters in the first block, with the number of filters doubling at each subsequent level, consistent with the contracting path of a 3D U-Net encoder.

Following hierarchical feature extraction, the classification head aggregates the resulting feature maps via Global Average Pooling. A Dropout layer (rate = 0.3) is subsequently applied for regularization, and the final prediction is produced through a dense layer with two output neurons and softmax activation.

### 3.2. Comparative Performance Analysis

In this section, the evaluation metrics of the proposed CNN are presented, derived from the repeated five-fold cross-validation strategy as detailed above. To assess the robustness and effectiveness of the proposed method, the same training procedure was applied to benchmark 3D CNN architectures widely used in volumetric medical imaging. Identical experimental conditions, including data splits, random seeds, and training settings, were maintained to ensure fair comparison. Additionally, statistical significance between the proposed model and the state-of-the-art models was assessed utilizing Wilcoxon Signed-Rank test. This non-parametric test was selected due to the paired nature of the cross-validation runs and the absence of normality assumptions, enabling robust comparison across the 15 fold-run results [47].

The benchmark architectures comprised a diverse set of 3D CNN models with a variety of depths and design strategies. Specifically, 3D ResNet10 and 3D ResNet50 were selected to represent lightweight and deeper residual network configurations, respectively. For both models, pre-trained weights from MedicalNet, obtained from training on 23 medical imaging datasets, were available [48]. Preliminary experiments were conducted in which the models were evaluated both from scratch and through fine-tuning using the pretrained weights, enabling adaptation to the classification task of this study. The fine-tuned variants demonstrated superior performance and were therefore selected for the final comparative evaluation.

The CNN architecture proposed by Chakrabarty et al. [12] was also included, as it addresses LGG/HGG classification among seven distinct classification tasks, in a segmentation-free framework. Finally, the 3D version of DenseNet121 provided by the MONAI framework was employed and trained from scratch, representing densely connected convolutional architectures in volumetric medical imaging [49,50]. For these architectures, no compatible publicly available pretrained volumetric weights were available.

#### 3.2.1. Internal Cross-Validation Results

Following the comparative evaluation framework described above, the internal repeated five-fold cross-validation results across all four cohorts are presented in Table 3. Mean values and the corresponding sample-based standard deviations are reported for ACC, F1-score, AUC, and MCC. Furthermore, statistical significance between models is indicated using the symbol corresponding to *p* < 0.001.

Across all evaluation metrics, she proposed architecture demonstrated superior mean performance compared to the evaluated models, accompanied by low standard deviation values, indicating stable behavior across folds. Pairwise Wilcoxon signed-rank comparisons provided supportive statistical evidence (*p* < 0.001) favoring the proposed model across all evaluation metrics relative to the benchmark architectures. Among the comparative models, 3D ResNet50 demonstrated the second-highest performance. The proposed CNN exceeded 3D ResNet50 by 2.7%, 1.9%, 2.2%, and 5.4% in ACC, F1-score, AUC, and MCC respectively.

To further evaluate model performance under the moderate class imbalance of the dataset, class-wise metrics were computed for the proposed CNN across the repeated five-fold cross-validation runs. Table 4 reports the mean and sample-based standard deviation for sensitivity (SENS), specificity (SPEC), precision (PREC), F1-score, and the Area Under the Precision–Recall Curve (AUPRC) separately for LGG and HGG cases. Both classes demonstrated high class-wise performance despite the moderate class imbalance, with slightly higher values for HGG cases. The AUPRC above 90% for the minority LGG class indicates satisfactory minority-class detection.

**Figure 4:**
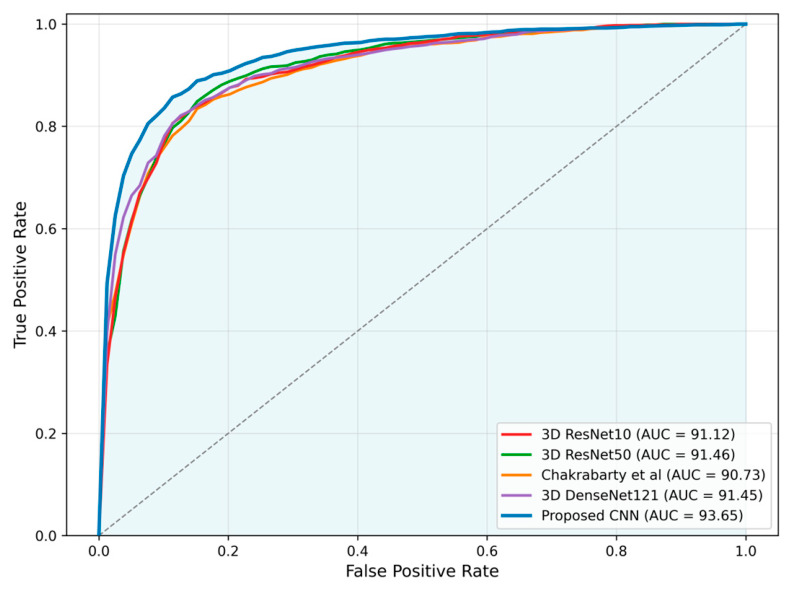
Comparison of the mean Receiver Operating Characteristic (ROC) curves for the proposed CNN and benchmark 3D architectures under internal LGG/HGG classification.

For the internal LGG/HGG classification task, the mean Receiver Operating Characteristic (ROC) curves, averaged across the 15 independent runs per model are depicted in Figure 4. The proposed architecture exhibits a consistently higher mean ROC curve relative to the benchmark models across most operating thresholds.

To address the possibility that dataset origin could act as a surrogate marker for tumor grade during internal validation, particularly due to the inclusion of the TCGA-LGG cohort, which includes only LGG cases, an additional dataset-stratified patient-level cross-validation analysis was performed. In this protocol, fold generation was stratified using the combined tumor-grade and dataset-origin label, thereby preserving both class and cohort distributions across folds. Table 5 presents the results of this stratification analysis across all validation metrics of the proposed CNN. The dataset-stratified patient-level protocol yielded comparable, and slightly improved, performance relative to the original patient-level stratified protocol. These findings suggest that the internal validation performance was not primarily driven by dataset-origin effects.

#### 3.2.2. External Validation on the REMBRANDT Dataset

To further evaluate the model’s robustness and generalization capability, an external validation experiment was conducted. During this phase, all CNNs were trained using the same protocol described for the repeated cross-validation procedure, while the REMBRANDT cohort was entirely excluded from training. Then, the best model obtained from each fold was used to generate predictions for all cases in the external cohort in the LGG/HGG classification task. Table 6 reports the performance metrics of the proposed CNN and the benchmark 3D architectures. Mean values along with the corresponding sample-based standard deviations are presented for ACC, F1-score, AUC, and MCC, while statistical significance is indicated by the corresponding symbols.

Regarding external evaluation, the proposed CNN again achieved the highest performance among the evaluated models across all metrics. Pairwise statistical comparisons indicated supportive evidence of improved performance across most metrics, whereas for the F1-score significance was observed only against one benchmark model. Among the comparative models, 3D DenseNet121 ranked second in ACC and F1-score, while 3D ResNet50 achieved the second-best performance in AUC and MCC. Additionally, the proposed CNN presented the lowest variability across all evaluation metrics, as reflected by the smallest standard deviation values among the compared models.

**Figure 5:**
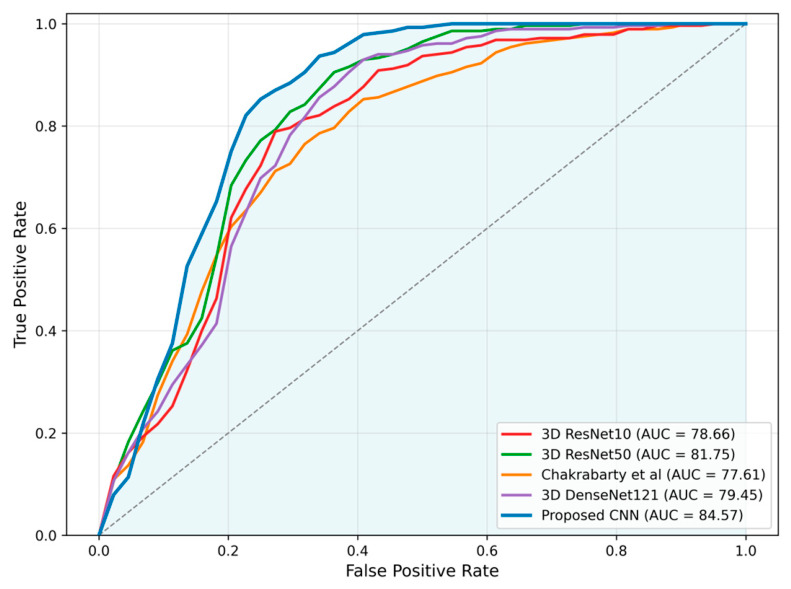
Comparison of the mean Receiver Operating Characteristic (ROC) curves for the proposed CNN and benchmark 3D architectures under external LGG/HGG classification on the REMBRANDT dataset.

Figure 5 illustrates the mean ROC curves of the proposed CNN and the comparative architectures for the external LGG/HGG classification task on the REMBRANDT dataset, averaged across the 15 independent runs. While benchmark models exhibited slightly higher sensitivity at very low operating thresholds, the proposed CNN achieved the highest AUC and maintained a higher ROC curve across the majority of the threshold range.

#### 3.2.3. Extended Multi-Cohort Hold-Out Validation

To further ensure the model’s robustness and account for institutional variations, an additional evaluation was performed using a 15% stratified hold-out test set selected across all four available data collections. A full leave-one-dataset-out cross-validation was precluded by the structural constraints of the available data. Specifically, the TCGA-LGG cohort is inherently limited to a single class (LGG only), while the EGD and UCSF-PDGM datasets constitute the core volume required for the segmentation-free CNN to learn generalized features. The resulting external hold-out set mitigates these limitations and includes 180 independent cases (67 LGG and 113 HGG).

**Figure 6:**
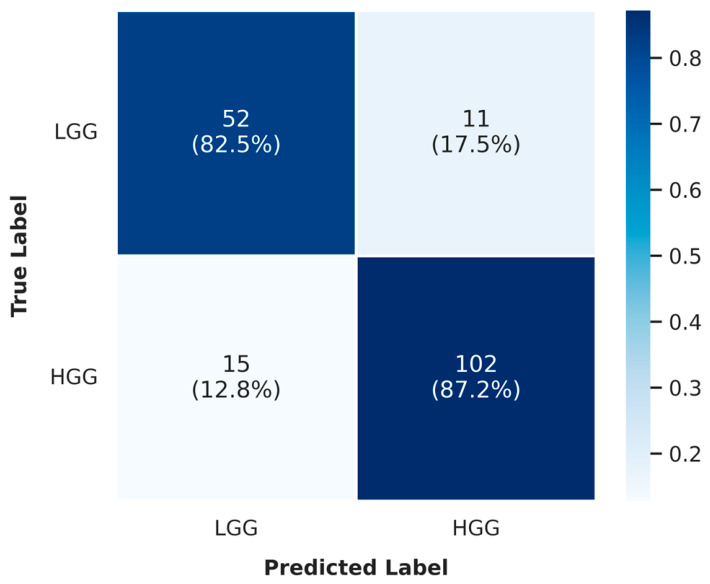
Mean confusion matrix across the 15-fold runs of the independent multi-cohort external hold-out validation, obtained using the proposed CNN.

The proposed CNN maintained high performance, achieving an overall accuracy of 85.56% ± 1.89% and a macro F1-score of 83.63% ± 1.60. Table 7 reports the mean values and corresponding standard deviations of the class-wise performance metrics obtained during the extended multi-cohort external hold-out validation. Additionally, Figure 6, illustrates the mean confusion matrix derived across all repeated cross-validation runs, providing a detailed description about predictions.

### 3.3. Ablation Study

#### 3.3.1. Architectural Component Analysis

An ablation study was conducted to evaluate the contribution of each component of the proposed methodology under the same internal validation protocol based on repeated five-fold cross-validation. Table 8 reports the mean performance and corresponding standard deviation for each configuration evaluated during the ablation analysis, including the 3D U-Net encoder baseline, the NAS-derived backbone CNN (U-Net–inspired), and the subsequent architectural enhancements (Residual and SE attention blocks), both individually and in combination.

Initially, the contracting path of the 3D U-Net was evaluated for the segmentation-free LGG/HGG classification, serving as the methodological starting point of the proposed approach. Subsequently, the NAS-derived backbone CNN demonstrated a substantial performance improvement compared with the 3D U-Net baseline, accompanied by statistically significant differences across all metrics. Additionally, the individual integration of residual connections and SE attention blocks into the backbone CNN was evaluated. When applied independently, neither component improved the backbone’s performance. Nevertheless, residual connections reduced performance variability, indicating improved model stability. The combined incorporation of both residual and SE mechanisms yielded the highest overall performance, forming the final proposed CNN architecture.

**Figure 7:**
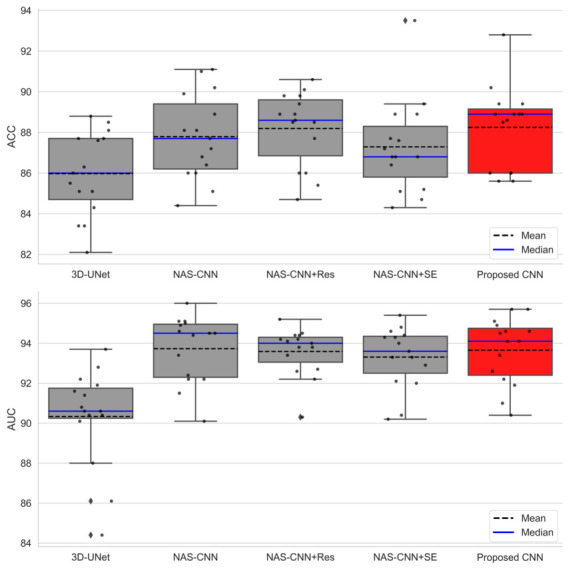
Distribution of model performance across the ablation study configurations over the 15 independent runs, illustrated using boxplots for ACC (**top**) and AUC (**bottom**). Black circles represent the individual runs, whereas black diamonds indicate outlier observations. Red color represents our proposed methodology.

The distribution of model performance across the ablation study is illustrated in Figure 7. Boxplots summarize the results of the 15 independent runs for each configuration in terms of ACC (top) and AUC (bottom).

#### 3.3.2. Missing-Modality Analysis

To investigate the contribution of MRI sequence availability, an additional ablation analysis was conducted using single-modality configurations of the proposed architecture. Specifically, the model was separately trained using only T1-CE or only FLAIR volumes and compared against the dual-modality configuration.

Furthermore, as the multi-center dataset included cases with incomplete MRI modalities, a sensitivity analysis was performed to evaluate the effect of the zero-filling strategy used for missing sequences. This approach examined the possibility of introducing bias through learning deterministic modality-availability patterns. In the analysis, the network was trained using only complete-modality patients, while modality-dropout training was applied on the training set, with dropout probabilities of {0.10, 0.15, 0.20}. In each run, one of the two MRI modalities was randomly masked with the corresponding probability in order to evaluate robustness against incomplete MRI protocols.

Table 9 summarizes the modality ablation and missing-modality sensitivity analysis results, presented as mean ± sample standard deviation, derived from the repeated 5-fold cross-validation experiments. The single-modality configurations demonstrated substantially lower performance compared with the dual-modality setting, highlighting the complementary contribution of T1-CE and FLAIR for glioma grading. Moreover, the complete-modality subset achieved higher performance than the full-cohort setting, while modality-dropout training further improved performance, with the *p* = 0.10 configuration achieving the highest overall metrics.

**Figure 8:**
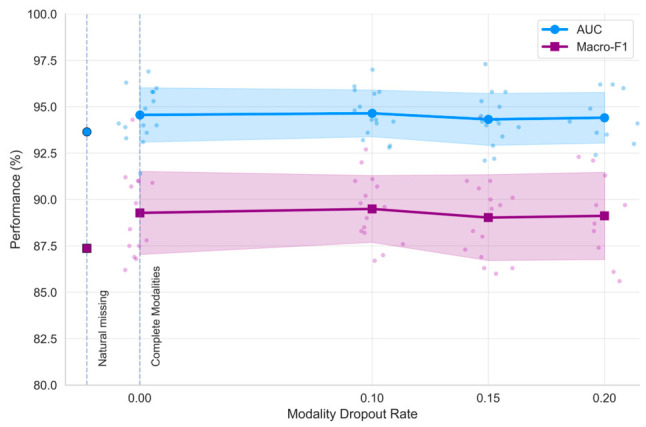
Comparative visualization of the missing-modality sensitivity analysis across modality-dropout configurations in terms of macro-F1 and AUC metrics. The dashed reference line indicates the full-cohort setting with natural missing modalities and zero-filling.

Figure 8 provides a comparative visualization of the missing-modality sensitivity analysis in terms of macro-F1 and AUC metrics across the evaluated modality-dropout configurations, including the full-cohort reference setting with natural missing modalities and zero-filling.

### 3.4. Grad-CAM—Model Interpretation

As a final stage of the present study, Grad-CAM was applied to provide visual explanations of the proposed model predictions on the LGG/HGG classification. Specifically, activation maps were extracted from the penultimate convolutional layer, since the final convolutional layer exhibited limited spatial resolution (15 × 15 × 11 voxels), which could result in low-quality visualization when projected onto the corresponding raw MRI images. Grad-CAM maps were generated for all validation samples from a representative fold-run of the proposed CNN, ensuring that explanations were derived exclusively from unseen data.

**Figure 9:**
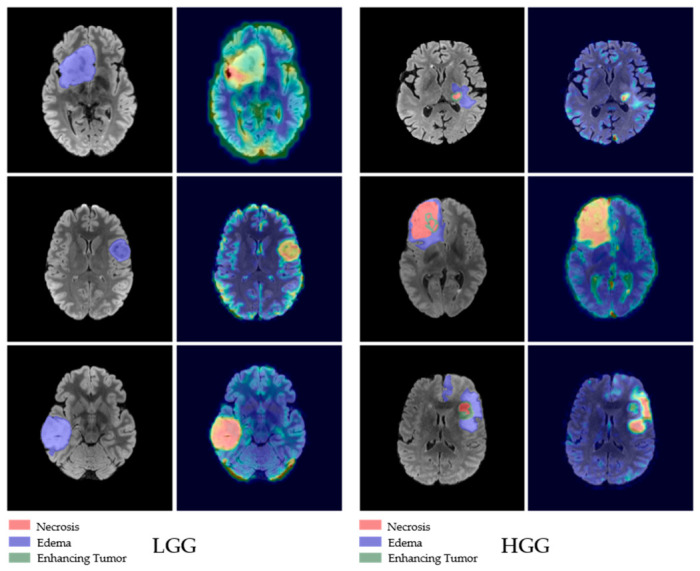
Qualitative visualization of three LGG and three HGG validation cases from the UCSF-PDGM dataset. For each category ground-truth tumor masks (**left**) are shown alongside the corresponding Grad-CAM activation maps (**right**) overlaid on the original axial MRI slices.

Figure 9 provides representative axial 2D visualizations of three LGG and HGG cases from the UCSF-PDGM dataset. For each case, the expert ground-truth tumor mask is shown on the left and the corresponding Grad-CAM activation overlay on the right. Regarding the tumor annotation, they include the subregions using the corresponding color coding. Grad-CAM activation maps are overlaid on the original MRI volumes, where blue indicates low contribution and red the highest activation levels, with intermediate colors representing progressively higher contributions.

Τhis qualitative analysis shows that the proposed CNN focuses on tumor-related regions when generating predictions, despite being trained in a segmentation-free manner. In LGG examples, activations are primarily concentrated around edema regions, whereas in HGG cases, the model demonstrated detailed focus on the whole tumor area, with strong activation patterns observed within necrotic regions.

**Figure 10:**
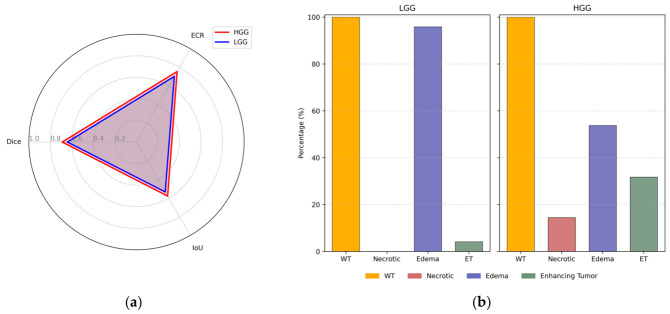
Quantitative Grad-CAM analysis in a representative cross-validation run: (**a**) Localization agreement between tumor masks and Grad-CAM activations for EGD and UCSF-PDGM cases in terms of DICE, IoU, and ECR for LGG and HGG. (**b**) Tumor subregion percentage contribution to the overall Grad-CAM activation for LGG and HGG UCSF-PDGM cases.

In addition to the qualitative analysis, a quantitative evaluation was conducted to provide a more comprehensive assessment of the proposed CNN, specifically regarding tumor localization. Figure 10a reports the mean localization metrics in terms of Dice, IoU and ECR for LGG (blue), and HGG (red) cases from the EGD and UCSF-PDGM validation cohorts, evaluated across 82 independent patients from the representative fold. The cohort-wide spatial agreement between the tumor masks and the 90th percentile of Grad-CAM activations yielded 0.67 ± 0.21 DICE, 0.56 ± 0.26 IoU, and 0.73 ± 0.20 ECR across both LGG/HGG predictions, with slightly improved localization observed for HGG cases. Figure 10b further illustrates the relative contribution of each tumor subregion to the model’s decision, presented as bar charts, summarizing the corresponding activation distribution within the UCSF-PDGM patient volumes. In HGG cases, increased contributions were observed in the necrotic and enhancing tumor subregions, whereas these regions showed minimal contribution in LGG cases.

### 3.5. Additional Glioma Classification Tasks Evaluation

The proposed CNN was derived from the LGG/HGG task-oriented NAS process and is based on a 3D U-Net–inspired architecture. An additional evaluation was conducted under the same validation scheme to examine whether the architecture could learn generalizable volumetric representations in a segmentation-free setting for related tasks, including IDH mutation prediction and multi-grade classification. Despite being designed for LGG/HGG classification, the proposed CNN achieved superior performance in IDH status prediction, while maintained satisfactory discrimination in the multi-grading classification task. A moderate decrease was observed in the F1-score for the multi-grading problem, reflecting the increased difficulty in distinguishing between LGG subclasses (Grade 2 and 3). Table 10 reports the mean values and corresponding standard deviations for both tasks.

**Figure 11:**
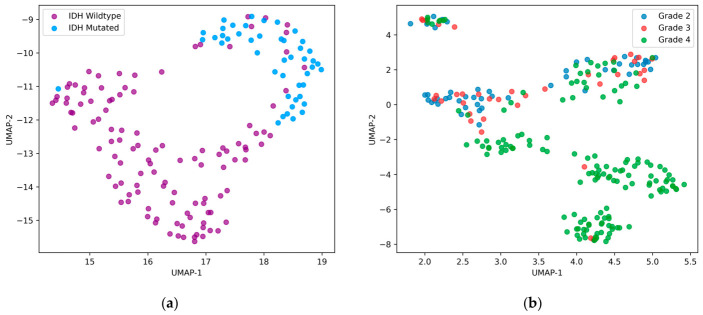
UMAP visualization of feature embeddings extracted from Global Average Pooling layer of the proposed CNN. (**a**) Embedding distribution for IDH mutation prediction; (**b**) Embedding distribution for multi-grade classification.

A visual representation of the learned feature embeddings for each classification task is presented in Figure 11. The embeddings were extracted from the Global Average Pooling layer of the proposed CNN from a representative fold-run and visualized using the Uniform Manifold Approximation and Projection (UMAP) dimensionality reduction framework. UMAP is a non-linear technique that projects high-dimensional data into a low-dimensional space while preserving local and global data structure [51]. Figure 11a illustrates the separation of MRI volumes according to IDH status, demonstrating clear clustering between mutant and wildtype cases. Figure 11b presents the corresponding visualization for tumor multi-grading, where separation between LGG and HGG is observed, although partial overlap between Grade 2 and Grade 3 samples appears.

## 4. Discussion

Numerous studies have reported high performance using 2D-based approaches for MRI glioma classification. However, these methods process individual slices independently, limiting their ability to capture the full volumetric context and increasing the risk of information leakage when slices from the same patient are distributed across training and testing sets [34,35,52]. In contrast, 3D models operate on whole volumes, preserving spatial consistency and enabling more reliable patient-level evaluation. Nevertheless, most existing 3D approaches rely on tumor segmentation as a prerequisite, introducing additional dependency on accurate annotations and potential error propagation to the classification task.

In the context of this study, the proposed CNN demonstrated strong classification performance, achieving a validation accuracy of 88.3% with a high ROC-AUC score of 0.936, indicating effective feature representation learning through volumetric processing without the need for expert annotations. External validation on the REMBRANDT cohort further confirmed the robustness of the approach, yielding 75.5% accuracy and a ROC-AUC of 0.846. Comparison with benchmark 3D architectures revealed notable performance differences, even under external validation, where pre-trained models typically benefit from improved initialization and enhanced generalization due to prior knowledge. However, the reduced external performance highlights the challenges associated with cross-dataset MRI heterogeneity and domain shift. To further assess cross-cohort robustness, an additional 15% multi-cohort hold-out evaluation was conducted, where the proposed CNN maintained competitive performance with an overall accuracy of 85.56% and a macro F1-score of 83.63%.

Although the absolute gains over benchmark models were moderate, the improvements remained consistent across evaluation metrics and may still be clinically meaningful in neuro-oncology applications, particularly in reducing the risk of tumor misclassification. Importantly, the additional NAS-related computational complexity was limited to the offline architecture search phase, while the final deployed model introduced no additional inference-time computational cost, while operating without prior use of tumor segmentation masks or expert annotations. These observations suggest the advantage of task-specific designed models via NAS, further supporting the notion that task-specific, data-driven architecture design, can achieve improved adaptability compared to general-purpose state-of-the-art models.

Nevertheless, due to the high computational cost of 3D CNNs, the optimization budget was limited to 50 trials. While this budget successfully identified a robust configuration, the NAS process represented a targeted rather than exhaustive exploration of the full architectural search space.

Furthermore, the missing-modality analysis suggested that zero-filled channels did not artificially inflate the reported performance, as the complete-modality subset achieved higher metrics than the full-cohort setting. Modality-dropout training further improved performance, indicating that random masking of MRI sequences may act as a robustness-oriented regularization strategy for incomplete multi-sequence MRI protocols.

In addition, the incorporation of XAI through Grad-CAM provided further insights into the model’s decision-making process. The resulting activation maps offered qualitative visualization of tumor regions, while quantitative agreement analysis with available ground-truth masks supported the spatial consistency of these activations. The model demonstrated consistent localization within tumor regions, including attention to relevant sub-regions that contribute to glioma discrimination. This observation supports the reliability of the proposed segmentation-independent framework and highlights its potential for enhanced interpretability in clinical settings. Nevertheless, the quantitative explainability assessment was conducted on a representative validation fold to provide an overall evaluation of the observed activation patterns.

Despite being designed for the LGG/HGG classification task, the proposed CNN was evaluated on IDH mutation prediction using a sufficiently representative subset of the dataset with available annotations. The model achieved even higher performance compared to the LGG/HGG task, indicating effective feature representation learning and strong generalization capability. However, this performance could potentially be influenced by underlying grade-associated characteristics, given the strong correlation between IDH mutation status and LGG cases. In addition, evaluation on multi-grade classification task (three classes) showed further insights into the model’s behavior. While the overall performance remained relatively high without significant degradation, a notable limitation was observed. Specifically, the discrimination between LGG subcategories was not satisfactory, which negatively impacted the corresponding F1-scores. These findings underscore that while the proposed framework is highly effective for its primary application of binary LGG/HGG classification, further refinement is required for reliable multi-grade stratification.

Beyond these task-specific observations, several broader limitations of the study should also be acknowledged. Although a relatively large cohort of 1194 patients was utilized, further improvements could be achieved with larger and more diverse datasets, particularly for volumetric processing. In addition, the retrospective nature of the data may limit the generalizability of the findings, while the incorporation of an independent dataset from a local clinical institution as external validation would further strengthen the reliability and clinical applicability of the proposed approach. Furthermore, variability across MRI acquisition protocols, scanner vendors, and public dataset annotations may affect the broader applicability of the proposed framework.

From a future perspective, extending the framework toward a unified multi-task learning setting represents a promising direction. Additionally, integrating clinically relevant molecular markers, such as 1p/19q co-deletion and MGMT promoter methylation, could further enhance the clinical value of the model, given sufficient annotated data. Moreover, future investigation of anatomically consistent augmentation strategies may further improve the robustness of the NAS-derived architecture under heterogeneous MRI acquisition settings.

Finally, Table 11 presents a comparative analysis of the proposed method with related approaches in the literature. The reported ACC and AUC values correspond exclusively to the glioma grading tasks, as provided in the respective studies. It should be noted that near-perfect ROC-AUC values reported in some works may not fully reflect class-wise performance, as indicated by lower F1-scores that provide a more realistic assessment. Furthermore, the presence or absence of tumor segmentation is considered to enable a more meaningful evaluation, although direct quantitative comparison across studies should still be interpreted cautiously due to methodological differences. In addition, the use of publicly available and/or institutional datasets is reported to enhance transparency. Under these conditions, the proposed method demonstrates competitive and consistent performance, particularly considering its segmentation-independent design.

## 5. Conclusions

In summary, this study introduces a segmentation-independent 3D CNN for glioma classification, incorporating NAS within a three-dimensional design space, an area that remains relatively underexplored in the literature. The proposed approach further benefits from targeted architectural enhancements that improve feature representation and overall model robustness. This approach contributes to the development of segmentation-independent volumetric classification frameworks without requiring expert-annotated tumor masks, while maintaining high performance. In addition, the emphasis placed on model transparency via XAI mitigates ‘black-box’ operation concerns and provides clinically meaningful insights into the decision-making process.

## Figures and Tables

**Table 1:** Final patient distribution across datasets after application of inclusion criteria, including information about glioma grading and availability of molecular IDH status.

Dataset	Total (N)	LGG	HGG	IDH Mutated	IDH Wildtype
REMBRANDT	68	49	19	-	-
TCGA-LGG	105	105	0	-	-
UCSF-PDGM	470	92	378	99	371
EGD	551	196	355	132	209
Total	1194	442	752	231	580

**Table 2:** Defined architectural search space for the NAS framework. The table summarizes the encoder structural hyperparameters and their corresponding search ranges.

Category	Hyperparameter	Search Range
Network Depth	Number of Blocks	3–6
Network Width	Number of Base Filters	16, 32
Block Structure	Convolutions per Block	1–3
Normalization	Normalization Type	Group, Instance
Normalization	Number of GN Groups	2, 4, 8, 16
Downsampling	Downsampling Strategy	Max Pooling, Average Pooling, Strided Convolution
Downsampling	First Block Downsampling	True, False
Classification Head	Head Type	Flatten *, Global Pooling **
Classification Head	Global Pooling Type **	Global Average Pooling, Global Max Pooling

* Applied when the number of blocks equals 3. ** Applied when the number of blocks is greater than 3.

**Table 3:** Performance comparison between the proposed CNN and benchmark 3D architectures under internal cross-validation. Results are reported as mean ± standard deviation.

Model	ACC	F1-Score	AUC	MCC
3D ResNet10 (MN)	84.52 ± 2.35 **^‡^**	84.39 ± 2.60 **^‡^**	91.12 ± 1.97 **^‡^**	67.14 ± 5.05 **^‡^**
3D ResNet50 (MN)	85.56 ± 2.10 **^‡^**	85.50 ± 2.16 **^‡^**	91.46 ± 2.17 **^‡^**	69.63 ± 4.10 **^‡^**
Chakrabarty et al.	84.83 ± 1.96 **^‡^**	83.77 ± 1.97 **^‡^**	90.73 ± 2.27 **^‡^**	68.16 ± 4.06 **^‡^**
3D DenseNet121	84.81 ± 1.69 **^‡^**	84.82 ± 1.72 **^‡^**	91.45 ± 1.86 **^‡^**	68.05 ± 3.88 **^‡^**
Proposed CNN	**88.25 ± 2.04**	**87.37 ± 2.29**	**93.65 ± 1.67**	**75.03 ± 4.46**

^‡^ Indicates *p* < 0.001 compared with the proposed CNN. Bold and underlined values indicate the best and second-best results, respectively.

**Table 4:** Class-wise internal cross-validation performance of the proposed CNN. Metrics are reported as mean ± sample standard deviation.

Class	SENS	SPEC	PREC	F1-Score	AUPRC
LGG	85.64 ± 6.24	91.33 ± 4.13	85.65 ± 5.73	85.43 ± 4.12	90.59 ± 4.31
HGG	91.33 ± 4.13	85.64 ± 6.24	91.71 ± 3.11	91.43 ± 2.34	96.14 ± 1.88

**Table 5:** Dataset-stratified sensitivity analysis results of the proposed CNN. Performance is reported for the original grade-stratified patient-level cross-validation and the grade-and-dataset-stratified patient-level cross-validation protocols. Metrics are presented as mean ± sample standard deviation.

Validation Protocol	ACC	F1-Score	AUC	MCC
Patient-level stratified	88.25 ± 2.04	87.37 ± 2.29	93.65 ± 1.67	75.03 ± 4.46
Dataset-stratified patient-level	**88.54 ± 1.82**	**87.55 ± 2.06**	**93.91 ± 1.37**	**75.34 ± 4.04**

Bold values indicate the highest results.

**Table 6:** Performance comparison between the proposed CNN and benchmark 3D architectures under external validation on the REMBRANDT cohort. Results are reported as mean ± standard deviation.

Model	ACC	F1-Score	AUC	MCC
3D ResNet10 (MN)	71.96 ± 5.29 **^†^**	72.76 ± 5.32	78.66 ± 3.25 **^‡^**	39.02 ± 12.69 **^‡^**
3D ResNet50 (MN)	70.98 ± 4.73 **^†^**	72.33 ± 4.58	81.75 ± 1.76 **^‡^**	47.11 ± 6.80 **^†^**
Chakrabarty et al.	69.71 ± 5.03 **^‡^**	67.05 ± 4.92 **^‡^**	77.61 ± 5.16 **^‡^**	39.79 ± 9.00 **^‡^**
3D DenseNet121	72.16 ± 4.43 **^†^**	73.36 ± 4.11	79.45 ± 2.35 **^‡^**	45.28 ± 5.93 **^‡^**
Proposed CNN	**75.51 ± 1.80**	**73.77 ± 2.02**	**84.57 ± 1.67**	**53.72 ± 5.27**

^†^ Indicates *p* < 0.05 compared with the proposed CNN. ^‡^ Indicates *p* < 0.001 compared with the proposed CNN. Bold and underlined values indicate the best and second-best results, respectively.

**Table 7:** Class-wise performance metrics of the extended multi-cohort external hold-out validation obtained using the proposed CNN. Results are reported as mean ± standard deviation.

Class	SENS	SPEC	PREC	F1-Score	AUPRC
LGG	77.95 ± 4.59	89.91 ± 3.57	82.51 ± 4.38	79.93 ± 1.90	85.67 ± 1.21
HGG	89.91 ± 3.57	77.95 ± 4.59	87.41 ± 1.96	88.56 ± 1.31	93.05 ± 0.51

**Table 8:** Performance comparison of the model configurations evaluated in the ablation study. Results are reported as mean ± standard deviation.

Architecture	Residual	SE	ACC	F1-Score	AUC	MCC
3D U-Net	-	-	85.97 ± 2.08	85.07 ± 2.15	90.33 ± 2.47	70.46 ± 4.10
NAS-CNN (baseline)	-	-	87.79 ± 2.09	86.84 ± 2.27	**93.73 ± 1.67**	74.04 ± 4.37
NAS-CNN	✓	-	88.19 ± 1.84	87.29 ± 2.18	93.59 ± 1.21	75.01 ± 4.07
NAS-CNN	-	✓	87.29 ± 2.31	86.31 ± 2.51	93.31 ± 1.55	72.61 ± 5.02
Proposed CNN	✓	✓	**88.25 ± 2.04**	**87.37 ± 2.29**	93.65 ± 1.67	**75.03 ± 4.46**

✓ indicates the presence of the corresponding architectural component. Bold and underlined values indicate the best and second-best results, respectively.

**Table 9:** Modality ablation and missing-sequence sensitivity analysis results obtained from repeated 5-fold cross-validation experiments, including single-modality configurations, complete-modality evaluation, and modality-dropout training. Results are reported as mean ± standard deviation.

Configuration	Strategy	ACC	F1-Score	AUC	MCC
Full Cohort	Natural missing + zero-filling	88.25 ± 2.04	87.37 ± 2.29	93.65 ± 1.67	75.03 ± 4.46
Complete-modality	No missing	90.53 ± 2.07	89.28 ± 2.24	94.56 ± 1.47	78.73 ± 4.47
T1-CE only	Single modality	79.35 ± 1.55	76.51 ± 2.27	86.51 ± 2.20	53.66 ± 4.10
FLAIR only	Single modality	78.36 ± 2.55	75.60 ± 2.85	83.98 ± 2.78	52.39 ± 5.27
Dropout (*p* = 0.10)	Random masking	**90.77 ± 1.58**	**89.49 ± 1.80**	**94.65 ± 1.26**	**79.11 ± 3.63**
Dropout (*p* = 0.15)	Random masking	90.32 ± 2.02	89.03 ± 2.32	94.32 ± 1.40	78.26 ± 4.71
Dropout (*p* = 0.20)	Random masking	90.68 ± 2.02	89.15 ± 2.44	94.41 ± 1.37	79.03 ± 4.52

Bold values indicate the best-performing results.

**Table 10:** Performance of the proposed CNN on additional glioma classification tasks, including IDH mutation prediction and multi-grade classification. Results are reported as mean ± standard deviation.

Classification Task	ACC	F1-Score	AUC	MCC
IDH Status	89.51 ± 2.21	87.33 ± 2.73	93.54 ± 2.38	74.96 ± 5.64
Multi-Grade	78.74 ± 1.35	58.78 ± 3.26	87.98 ± 1.92	58.88 ± 2.21

**Table 11:** Comparative overview of related studies for glioma classification performance. Reported metrics correspond to the classification performance as presented in each study. Dataset indices are provided below.

Study	Year	Input	Segmentation	ACC AUC	Datasets
[34]	2024	2D	Not required	98.56% 0.999	3 (^9,11,13^)
[7]	2021	3D	Required	90.00% 0.933	5 (^2,5,8,12,13^)
[8]	2025	3D	Required	89.35% 0.957	8 (^4,7,8,10,12,13,14,15^)
[12]	2021	3D	Not required	- 0.98, 0.99 (LGG, HGG)	6 (^1,2,8,12,13,16^)
[11]	2025	3D	Not required	86.4% 0.892	6 (^3,4,6,8,9,14^)
Proposed Method	2026	3D	Not required	88.25% 0.937	4 (^4,9,13,14^)

^1^ BraTS-2018, ^2^ BraTS-2019, ^3^ BraTS-2020, ^4^ EGD, ^5^ GUH, ^6^ GZPH, ^7^ Ivy Gap, ^8^ LGG-1p19q, ^9^ REMBRANDT, ^10^ RHUH-GBM, ^11^ RIDER, ^12^ TCGA-GBM, ^13^ TCGA-LGG, ^14^ UCSF-PDGM, ^15^ UPenn-GBM, ^16^ WUSM.

## Data Availability

The datasets analyzed in this study are publicly available from the corresponding repositories, subject to terms and conditions defined by each repository. Specifically, data were obtained from The Cancer Imaging Archive, TCIA [13] (REMBRANDT, TCGA-LGG, and UCSF-PDGM collections) and from Erasmus Glioma Database [17] (EGD collection), which are available through their respective data access portals.

## Abbreviations

The following abbreviations are used in this manuscript:

ACC	Accuracy
AUC	Area Under the Curve
AUPRC	Area Under the Precision–Recall Curve
CAD	Computer-Aided Diagnosis
CNN	Convolutional Neural Network
CNS	Central Nervous System
ECR	Energy Concentration Ratio
FLAIR	Fluid-Attenuated Inversion Recovery
Grad-CAM	Gradient-weighted Class Activation Mapping
HGG	High-Grade Glioma
IDH	Isocitrate Dehydrogenase
IoU	Intersection over Union
LGG	Low-Grade Glioma
MCC	Matthews Correlation Coefficient
MRI	Magnetic Resonance Imaging
NAS	Neural Architecture Search
ROC	Receiver Operating Characteristic
ROI	Region of Interest
SE	Squeeze-and-Excitation
T1-CE	T1-weighted Contrast-Enhanced
TPE	Tree-structured Parzen Estimator
WHO	World Health Organization
XAI	Explainable Artificial Intelligence

## Appendix A

**Table A1:** Architectural characteristics of the top three NAS-derived 3D CNN configurations identified during the search process.

Hyperparameter	Rank-1	Rank-2	Rank-3
Number of Blocks	4	6	5
Number of Base Filters	16	32	16
Convolutions per Block	1	1	1
Normalization Type	Group	Group	Instance
Number of GN Groups	8	8	-
Downsampling Strategy	Max Pooling	Max Pooling	Max Pooling
First Block Downsampling	False	True	True
Head Type	Global Pooling	Global Pooling	Global Pooling
Global Pooling Type	Global Max Pooling	Global Max Pooling	Global Average Pooling
